# Placing the tibial component of an ankle prosthesis: results of a CT-based simulation in healthy adults

**DOI:** 10.1007/s00590-024-04112-4

**Published:** 2024-09-27

**Authors:** Patrick Gahr, Josephine Wittmüß, Heiner Martin, Thomas Beyer, Dagmar-C. Fischer, Thomas Mittlmeier

**Affiliations:** 1 Department of Trauma, Hand and Reconstructive Surgery, Rostock University Medical Center, Schillingallee 35, 18057 Rostock, Germany; 2 Institute for Biomedical Engineering, Rostock, Germany; 3 Department of Pediatrics, Rostock University Medical Center, Rostock, Germany; 4 Institute of Diagnostic and Interventional Radiology, Pediatric Radiology and Neuroradiology, Rostock University Medical Center, Rostock, Germany

**Keywords:** Ankle joint, Ankle arthroplasty, Ankle replacement, Distal tibia, Computed tomography, Aseptic loosening

## Abstract

**Purpose:**

To characterize the 3D geometry of the distal tibia resection area from healthy individuals using CT-based digital implantation for proper preoperative sizing of TAA tibia component placement.

**Methods:**

Standardized CT images of healthy ankle joints serving as intra-individual references for treatment of contralateral injuries were identified. The tibial cross section dedicated to virtually host the tibial component was digitally prepared, and the size of the virtual contact surface was calculated. Finally, out of five prototypes the one fitting best in terms of size and alignment was identified.

**Results:**

CT scans taken from 319 subjects were used for the virtual implantation procedure. Body height and size of the distal tibia contact area correlated (*r* = 0.49 and 0.42 in females and males, each *p* < 0.001). Prosthesis sizes 2 and 3 fit well for the vast majority of patients, while the smallest and largest sizes are rarely required.

**Conclusions:**

Digital implantation of the tibial component should be considered a valuable tool for preoperative planning as well as for the development of new implant types.

## Introduction

In patients with osteoarthritis, total ankle arthroplasty (TAA) has emerged as a viable alternative to arthrodesis [1–3], despite the inherent challenges of its technique. These challenges primarily stem from the limited range of motion, necessitating continuous efforts to refine surgical techniques and enhance implant designs. For most implant designs, placing the tibial component of the prosthesis requires a planar resection of the tibia without removal of the anterior cortex as this is essential for stable support. In case of the Hintermann H3™ total ankle replacement system only 2–3 mm of the tibia have to be resected and, contrasting to the initial recommendations, neither cementation nor screw fixation is required [4, 5]. Instead, the primary stability and secondary stability of the tibial component strongly rely on a sufficient bone stock and proper implant placement, i.e., fitting between the shape and area of both surfaces. Inadequate positioning and/or sizing of the prosthesis may cause impingement (“gutter pain”) and migration with the inherent risks of stress shielding, formation of periprosthetic cysts and finally loosening of the prosthesis [4]. Thus, a careful pre-operative planning in terms of the resection planes as well as the shape, size and exact positioning of the implant is required.

We hypothesized that X-ray computed tomography (CT) images of the ankle will not only be suited to simulate tibial resection and implantation of the tibial component of an ankle replacement system but will allow to identify the tibial component with the best fit in terms of size and positioning as well. A prototypic modular total ankle replacement prosthesis was used to investigate these issues via bilateral CT scans which were routinely taken for comparison of the distal tibiofibular joints in male and female patients suffering from an isolated ankle fracture.

## Methods

We conducted a theoretical–experimental study based on radiologic imaging. CT images taken between January 2006 and December 2016 from both ankles of patients undergoing isolated ankle fracture surgery were retrieved from our clinical information system. Images from the contralateral leg were eligible, if (i) the ankle joint was not affected, (ii) a positioning device was used to guarantee a standardized view of the ankle joint, (iii) a complete scan of the ankle joint was available and (iv) an intact inferior tibial articular surface (facies articularis inferior tibiae) was present. The detection of any lesion affecting the inferior articular surface or the medial malleolus led to the exclusion of the respective set of data. Prior to pseudonymization of the images, anthropometric data (age at the time of the examination, gender, height and weight) were retrieved by chart review.

### Analysis of the CT scans with virtual implantation of the tibial component

**Fig. 1:**
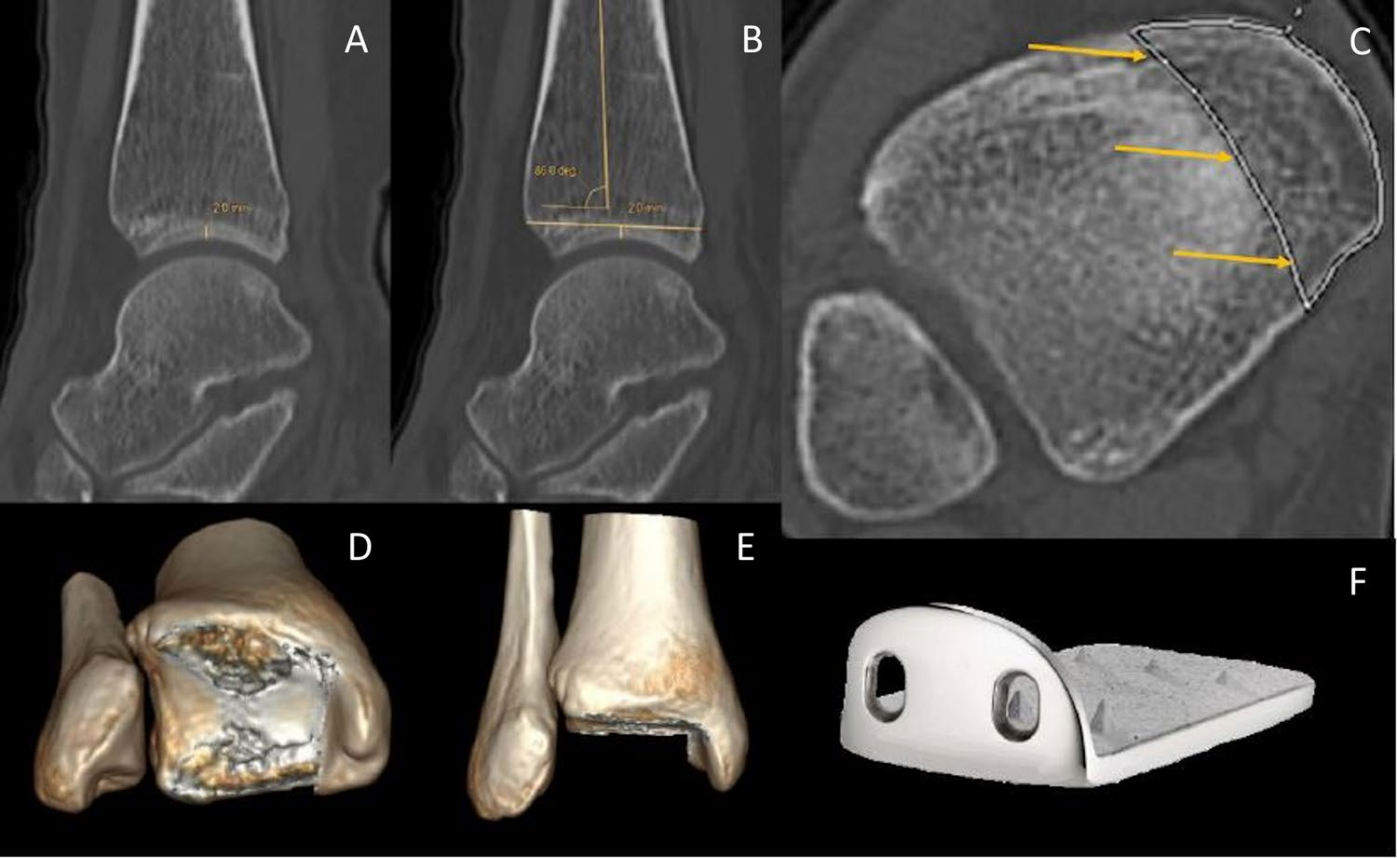
Work flow for generation of the tibial surface area dedicated to host the implant (**A–C**) together with a 3D visualization of the resection plane (**D**, **E**) and a representation of the prosthesis type (**F**). Landmarks defining height (**A**) and orientation (**B**) of the resection area together with a transversal view of the resection area (**C**). The arrows indicate the lateral border of the medial malleolus which should not be affected by the implantation procedure. The margins of the medial malleolus were digitally transferred from three different coronal sections and marked on the transversal plane prior to subjecting the image to the CAD system. There are ventral (D) and distal (E) views of the resection area after 3D reconstruction together with a representation of the prosthesis (F)

**Fig. 2:**
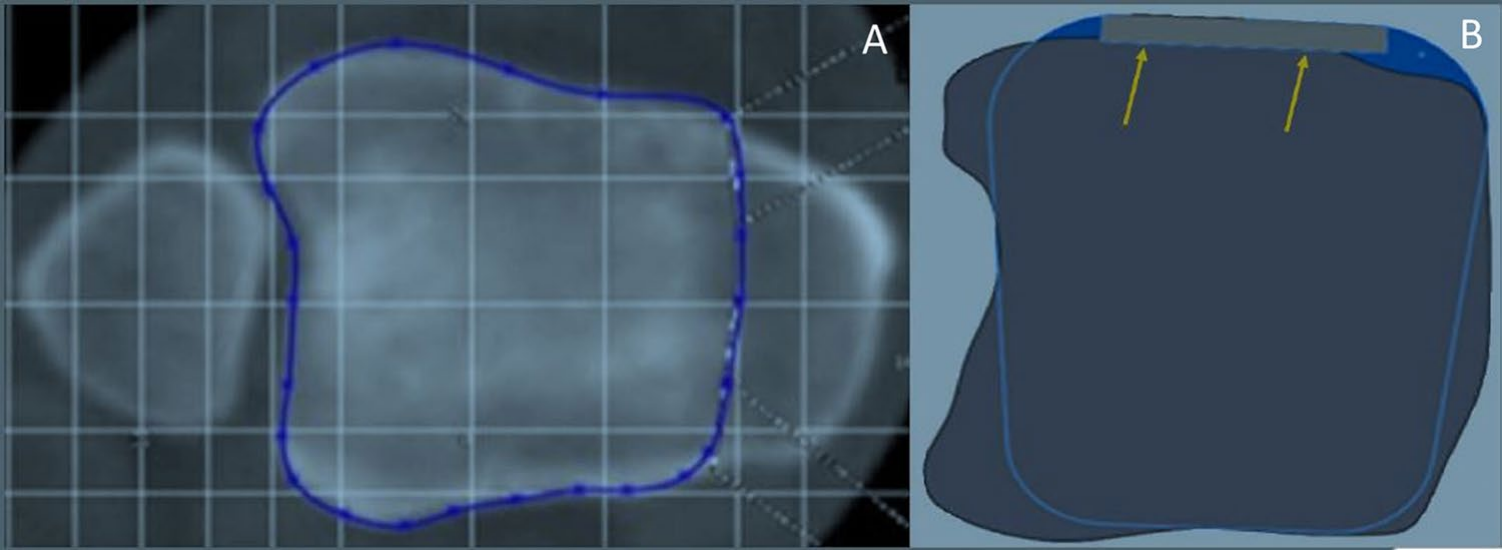
Fitting of the tibial resection plane for calculation of the area size (**A**) and positioning of the tibial component (**B**) with minimal overlap and parallel alignment of the ventral prosthetic shield (arrows)

Prototypes of an ankle joint prosthesis with 5 different modular sizes for the tibial component corresponding to surface areas of 884.40 mm^2^ (size 1), 1028.60 mm^2^ (size 2), 1178.50 mm^2^ (size 3), 1403.60 mm^2^ (size 4) and 1600.80 mm^2^ (size 5) were provided by OHST Medizintechnik AG, Rathenow, Germany. Independent of the prosthesis size, implantation requires a resection plane 2 mm proximal to the dome of the distal tibial articular surface with 4° posterior inclination corresponding to the posterior slope. Thus, we performed sagittal reconstructions (Aquarius iNtuition Edition Version 4.4.13.P2, TeraRecon Inc., Durham, NC 27703, USA), identified the apex of the tibial joint surface and simulated the transverse osteotomy on a paraxial cross section with 4° posterior inclination. The margins of the medial malleolus were transferred from three different coronal sections and marked on the transversal plane prior to subjecting the image to a CAD system (SolidWorks Premium 2013 × 64 Edition; Dassault Systèmes SolidWorks Corporation, Vélizy-Villacoublay, France) for simulation of the implantation procedure using either one of the available prototypes. In particular, the shape and the area of either surface were approximated by spline interpolation and numeric integration. Criteria for an optimal fit between the tibial transverse surface and the prosthesis were minimal overlap, a maximum of cortical contact and the parallel alignment of the ventral prosthetic shield (Figs. 1A–F and 2A, B).

### Statistical analysis

All data were summarized in a spreadsheet (Microsoft® Excel Version 16.6, Microsoft Corporation, USA). For visualization and statistical evaluation, Sigma Plot 13 (Systat Software GmbH, Germany) and SPSS Software package version 27 (SPSS GmbH, Germany) were used. Descriptive statistics were computed for continuous variables, and the Kolmogorov–Smirnov test was used to check normal distribution. Results are given as mean ± SD or median and range, as appropriate. Data were categorized according to sex and the unpaired Student’s *t* test (two-tailed) or the nonparametric Wilcoxon signed rank test was used as appropriate and correlation between normally and non-normally distributed variables was investigated according to Pearson or Spearman, respectively. All p-values are two-sided, and a p-value below 0.05 was considered significant.

## Results

We retrieved 436 CTs from the clinical information system, and 319 of those were deemed eligible. The anthropometric characteristics of the study population are summarized in Table 1.

**Table 1:** Anthropometric and demographic data of the study population

	Female (*n* = 137)	Male (*n* = 182)	*p*
Age [year]	54.0 (15.0–81.0)	43.5 (16.0–89.0)	< 0.001
Height [cm]	165.0 (146.0–180.0)	180.0 (162.0–203.0)	< 0.001
Weight [kg]	75.0 (44.0–140)	85.0 (55.0–155)	< 0.001
BMI [kg/m^2^]	23.4 (13.3–43.2)	23.7 (15.6–38.8)	*p* = 0.059

**Fig. 3:**
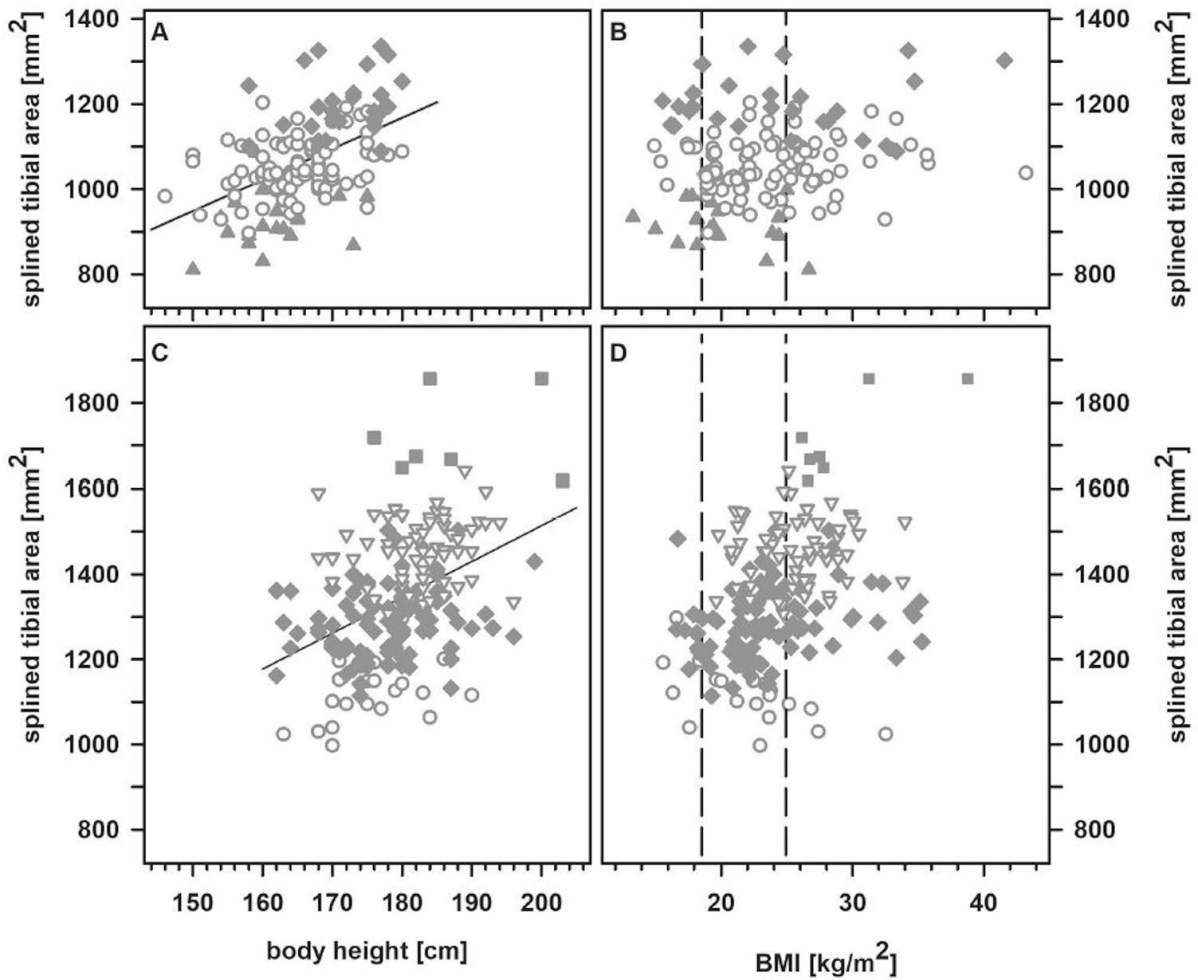
Association between the size of the resection area and body height (**A**, **C)** and BMI (**B**, **D**) in females (**A**, **B**) and males (**C**, **D**). Symbols indicate the corresponding size of the prostheses showing optimal fit (size 1: filled triangle up; size 2: open circle; size 3: filled diamond; size 4: open triangle down; size 5: filled square). The lower and upper limits for a physiological BMI are indicated by vertical dotted lines

Per subject, the transverse tibial plane dedicated to host the prosthesis was prepared and all of these images were used for virtual implantation of the prosthesis, i.e., calculation of the surface area by means of a spline function and identification of the best fitting prototype with respect to size and orientation. The size of the tibial surface dedicated to host the prosthesis ranged from 997.3 to 1856.7 mm^2^ in males (mean 1341.2 mm^2^, SD 153.0 mm^2^) and 810.3 to 1335.3 mm^2^ in females (mean 1064.0 mm^2^, SD 102.9 mm^2^), respectively. The size of the resection surface dedicated to host the prosthesis is related to the body height (*r* = 0.49 and *r* = 0.42 in females and males; each *p* < 0.001) but virtually not to the BMI (Fig. 3).

**Fig. 4:**
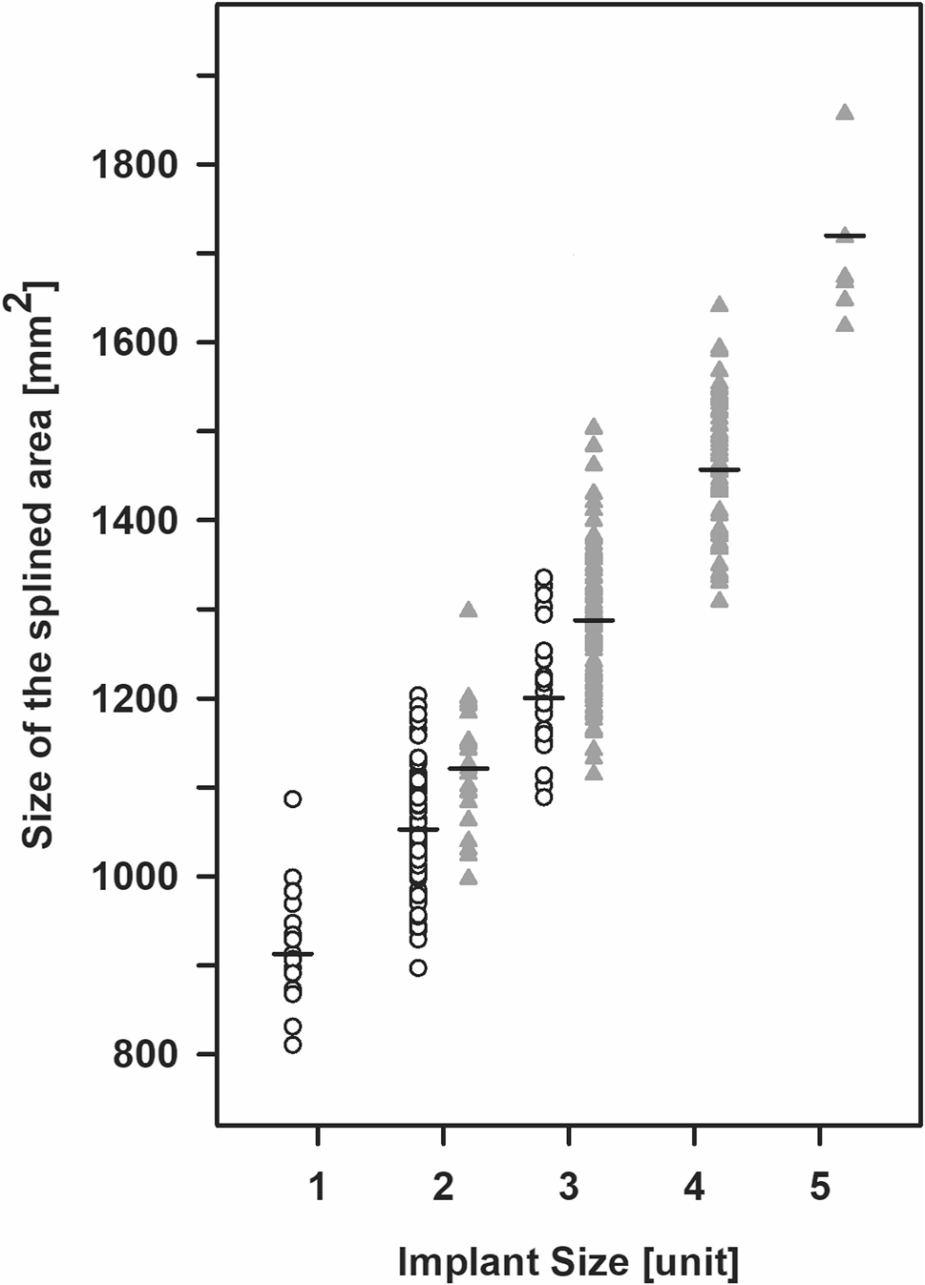
Relationship between tibial contact area and selected implant size in males (filled triangle up) and females (open circle)

Subsequently, we were able to determine minimum and maximum sizes of this area per size of the prosthesis. Size 3 showed the widest range (414.8 mm^2^), which extended from 1088.7 to 1503.5 mm^2^ and overlapped with all other sizes except for size 5, which covered the smallest range of application (238.5 mm^2^). There was neither overlap between the ranges of size 5 with sizes 1 to 3 nor between sizes 1 and 4 (Fig. 4).

Thus, within the entire group prosthesis components of size 2 (112 out of 319) and 3 (120 out of 319) were selected most frequently. By contrast, prostheses of sizes 1 and 5 were exclusively chosen for females (19 out of 137) and males (7 out of 182), respectively. Furthermore, size 2 of the prosthesis was chosen for almost two-thirds (91 out of 137) of the scans taken from females, while size 3 was selected for more than half of the cross sections (93 out of 182) obtained from males.

## Discussion

Total ankle replacement is technically demanding and the generally low case load results in a flat learning curve. Consequently, results reported by single centers are usually better and complication rates lower than those reported by registry studies [6]. The most common complications after TAA are mechanical problems such as implant subsidence, aseptic loosening and intraoperative periprosthetic fractures. These events carry a medium to high risk of ankle replacement failure [4]. While talar subsidence was one of the most common reasons for revision in earlier-generation prostheses, with current systems loosening of the tibial component turned out to occur with a five times higher frequency compared to the talar component [7].

Several studies investigated the adverse effects of implant malpositioning on the functional outcome and the rate of mechanical complications after TAA [5, 8–10]. In summary, rotational alignment as well as anteroposterior positioning of the tibial component turned out to be crucial for long-term stability.

Digital implantation based on an axial cross section allowed us to find the optimal size and anteroposterior positioning via identification of a balance between maximum cortical contact area and minimum overlap of the components. Of note, the proposed procedure for generation of the transverse cross section dedicated to host the tibial component can be adopted to simulate implantation of other prosthesis systems, as well. This is in line with the trend toward patient-specific instrumentation (PSI) and custom-made cutting guides based on preoperative CT templating [11]. In addition, some authors recommend CT scan under weight-bearing conditions (WBCT) for better reconstruction of the mechanical axis [12].

Long-term biomechanical stability of anchoring the tibial component essentially relies on the contact with the tibial cortical rim providing the highest stability of the cross-sectional area [13]. Thus, the shape of the implant, i.e., the congruency between the tibial and implant contact areas has to be considered as well. In that regard, our approach not only enables to compare the size of either surface but to rotate the implant according to the above-mentioned needs.

For the vast majority of men and women, however, sizes 2 and 3 will be appropriate [14]. Therefore, it does not seem necessary to develop off-the shelf implants in extremely small or large sizes. In addition, the patient´s height should be taken into account due to the strong correlation with the size of the distal tibial contact area. This information, which is based on a large group of healthy adults, can serve as a rough guide for size selection if no preoperative CT scan is available.

### Limitations of the study

Although one may consider the utilization of only one type of implant as a limitation, we are convinced that our approach is easily adaptable to other implant geometries. However, if the anchoring of the tibial component is based on a central keel or similar components, the selected two-dimensional simulation based on the axial section alone is not sufficient. Multiplanar reconstructions are then required to enable three-dimensional planning. The digital implantation, oriented on an axial cross section, allowed us to find the optimal rotation for the best cortical contact, i.e., the parallel alignment between the anterior surface of the distal tibia and the ventral prosthesis shield, on the one hand, and the circumferential cortical support of the implant, on the other. However, our method does not allow any conclusions to be drawn about the quality of the rotation of the entire joint replacement, as this would require the integration of the ankle joint's axis of rotation.

## Conclusions

Digital segmentation based on paraxial CT cross sections at the level of the resection plane is a valuable technique for the dimensioning and positioning of tibial components in ankle replacement. The method is suitable for preoperative planning. For modular systems, this technique allows to select the component best fitting to the anatomical conditions. Furthermore, the data will help to develop and size new implant types as the inter-individual variability in the size of the tibial cross-sectional area is relatively low and correlates with the patient's height. Especially, medium-sized implants fit more than two-thirds of patients. This finding may also be a clinically valuable additional tool for pre- and intraoperative size matching of tibial components.

## Data Availability

The dataset analyzed during the current study is available from the corresponding author on reasonable request.

## References

[1] Terrell RD, Montgomery SR, Pannell WC, Sandlin MI, Inoue H, Wang JC, SooHoo NF (2013) Comparison of practice patterns in total ankle replacement and ankle fusion in the United States. Foot Ankle Int 34(11):1486–1492 23774468 10.1177/1071100713494380

[2] Haddad SL, Coetzee JC, Estok R, Fahrbach K, Banel D, Nalysnyk L (2007) Intermediate and long-term outcomes of total ankle arthroplasty and ankle arthrodesis. A systematic review of the literature. J Bone Joint Surg Am 89(9):1899–905 17768184 10.2106/JBJS.F.01149

[3] Goldberg AJ, Chowdhury K, Bordea E, Hauptmannova I, Blackstone J, Brooking D et al (2022) Total ankle replacement versus arthrodesis for end-stage ankle osteoarthritis: a randomized controlled trial. Ann Intern Med 175(12):1648–1657 36375147 10.7326/M22-2058

[4] Glazebrook MA, Arsenault K, Dunbar M (2009) Evidence-based classification of complications in total ankle arthroplasty. Foot Ankle Int 30(10):945–949 19796587 10.3113/FAI.2009.0945

[5] Hintermann B, Susdorf R, Krahenbuhl N, Ruiz R (2020) Axial rotational alignment in mobile-bearing total ankle arthroplasty. Foot Ankle Int 41(5):521–528 31996033 10.1177/1071100720902838

[6] Mittlmeier T (2013) Arthrodesis versus total joint replacement of the ankle. Unfallchirurg 116(6):537–50 23744178 10.1007/s00113-013-2366-5

[7] Cody EA, Bejarano-Pineda L, Lachman JR, Taylor MA, Gausden EB, DeOrio JK, Easley ME, Nunley JA 2nd (2019) Risk factors for failure of total ankle arthroplasty with a minimum five years of follow-up. Foot Ankle Int 40(3):249–258 30345818 10.1177/1071100718806474

[8] Cenni F, Leardini A, Cheli A, Catani F, Belvedere C, Romagnoli M, Giannini S (2012) Position of the prosthesis components in total ankle replacement and the effect on motion at the replaced joint. Int Orthop 36(3):571–578 21789498 10.1007/s00264-011-1323-6 PMC3291768

[9] Najefi AA, Ghani Y, Goldberg A (2019) Role of rotation in total ankle replacement. Foot Ankle Int 40(12):1358–1367 31402689 10.1177/1071100719867068

[10] Saito GH, Sturnick DR, Ellis SJ, Deland JT, Demetracopoulos CA (2019) Influence of tibial component position on altered kinematics following total ankle arthroplasty during simulated gait. Foot Ankle Int 40(8):873–879 31244338 10.1177/1071100719858620

[11] Townshend D, Bing A, Blundell C, Clough T, Davenport J, Davies H, Davis J, Dhar S, Hepple S, Kakwani R, Karski M, Makwana N, McKinley J, Murty A, Raglan M, Shalaby H, Sharpe I, Smith R, Taylor H, Goldberg A (2023) Two to five-year outcomes of total ankle arthroplasty with the infinity fixed-bearing implant: a concise follow-up of a previous report. J Bone Joint Surg Am 105(23):1846–1856 38063779 10.2106/JBJS.22.01294 PMC10695343

[12] Thompson MJ, Consul D, Umbel BD, Berlet GC (2021) Accuracy of weightbearing CT scans for patient-specific instrumentation in total ankle arthroplasty. Foot Ankle Orthop 6(4):24730114211061492 35097485 10.1177/24730114211061493 PMC8664310

[13] Hintermann B, Pitner M-T, Grabmayr S, Hintermann B (2005) Endoprothetik des Sprunggelenks : historischer Überblick, aktuelle Therapiekonzepte und Entwicklungen Wien etc. Springer

[14] Penner M, Davis WH, Wing K, Bemenderfer T, Waly F, Anderson RB (2019) The infinity total ankle system: early clinical results with 2- to 4-year follow-up. Foot Ankle Spec 12(2):159–166 29865886 10.1177/1938640018777601 PMC6507063

